# Associations between unhealthy dieting behaviors and tobacco use among adolescents

**DOI:** 10.1186/s40337-016-0126-y

**Published:** 2016-12-15

**Authors:** Megan E. Sutter, Aashir Nasim, Susan Veldheer, Caroline O. Cobb

**Affiliations:** 1 Department of Psychology, Virginia Commonwealth University, 808 West Franklin St, Richmond, VA 23284 USA; 2 Department of African American Studies, Virginia Commonwealth University, Richmond, VA USA; 3 Department of Public Health Sciences, Penn State College of Medicine, Hershey, PA USA; 4 Center for the Study of Tobacco Products, Virginia Commonwealth University, Richmond, VA USA

**Keywords:** Smoking & tobacco, Child & adolescent health, Nutrition & diet, Risk behaviors

## Abstract

**Background:**

Cigarette smoking is an important risk factor for unhealthy dieting behaviors (UDBs) in youth. The role of alternative tobacco products and UDB engagement has yet to be examined empirically despite concerning trends in use. This study aimed to examine UDB prevalence in a U.S. geographic region-specific adolescent sample and associations with a variety of tobacco use behaviors and perceptions.

**Methods:**

Weighted data from the 2013 Virginia Youth Survey were analyzed (*n* = 6903). UDBs assessed included past 30-day fasting, diet pill use, and vomiting/laxative use. Tobacco-related items were ever and past 30-day cigarette smoking, past 30-day smokeless tobacco and cigar use, and the perception that smokers have more friends. UDB prevalence was recoded by the number of behaviors endorsed (0, 1, and 2+). Bivariate and multinomial regression models were used to examine associations between covariates and number of UDBs endorsed by gender.

**Results:**

Overall, nearly 16% engaged in at least one UDB. Fasting was most prevalent (14.2%) followed by vomiting/laxative (7.0%) and diet pill use (6.1%). Across gender, ever cigarette smoking, past 30-day cigar use, and the perception that smokers have more friends were positively associated with UDB engagement in relative isolation as well as in combination.

**Conclusions:**

Findings highlight the importance of tobacco-related factors for weight control behaviors and are the first to identify an association between UDB incidence and an alternative tobacco product, cigars. This work should inform prevention efforts for tobacco use and UDBs and underscores the need to address the use of any tobacco for weight control.

## Plain English Summary

Smoking cigarettes in order to control weight has been reported frequently among youth. Indeed, smoking is a risk factor for unhealthy dieting behaviors, such as vomiting, using diet pills, and simply not eating. Until now, the links between other types of tobacco products like cigars and snuff have not been looked at in connection with unhealthy dieting behaviors. This study found that having ever smoked cigarettes, recent use of cigars, little cigars, or cigarillos, as well as perceived social benefits of smoking were linked to unhealthy dieting behaviors. These findings highlight the importance of tobacco use, including both cigarettes and various forms of cigars, in the role of weight control among youth and should be a focus for prevention interventions in the future.

## Background

While smoking rates in 2014 among United States (U.S.) adolescents reached their lowest in the past 40 years, 14% of youth aged approximately 17–18 years (i.e., high school seniors) still report past month smoking, and 87% of adult smokers initiate smoking before age 18 [1, 2]. Importantly, smoking and related health disparities are still major issues in the U.S. particularly in light of concerning trends in the use of other tobacco products among youth (e.g., electronic cigarettes, hookah, little cigars/cigarillos) [2, 3]. For example past 30-day electronic cigarette use exceeded all other tobacco use types for U.S. students aged typically 11–13 years (middle school; 4%) and 14–18 years (high school; 13%) students in 2014 [3]. In Virginia (U.S. south-eastern geographic region), past 30-day cigarette smoking and cigar, cigarillo, or little cigar prevalence was equivalent (11% for both in 2014) [4]. These data demonstrate a need to better understand the factors influencing all types of tobacco use during this vulnerable age period [2].

One particular factor that may impact tobacco use in adolescence is the weight controlling effects of nicotine [5]. Biologically, nicotine mediates the association between cigarette smoking and body weight by increasing the resting metabolic rate of tobacco users, while smoking behaviorally provides an alternative for eating and decreasing caloric intake, together resulting in reduced body weight among tobacco users [5]. To date, studies have primarily focused on cigarette smoking as the tobacco product (i.e., nicotine delivery system) of interest. Weight concerns in grade 8 (e.g., middle school) and 11 (e.g., high school) were found to predict cigarette smoking status in young adulthood in adolescent females in a prospectively measured Canadian-based sample [6]. In this same sample, increased BMI at these grades predicted cigarette smoking among males [6]. Adolescent smokers also tend to report more unhealthy dieting behaviors (UDBs; e.g., fasting, use of laxatives, diuretics, diet pills, or vomiting), which have been attributed to the expectancy to control one’s weight, particularly among heavy smokers [7, 8]. In one U.S. geographic region-specific sample, adolescents who engaged in any UDBs were more likely to report cigarette use compared to those who did not report UDBs in 6th, 9th and 12th grades (correspond to middle and high school levels) [9]. It is unclear what proportion of cigarette smokers initiate tobacco use as a form of weight control, but there is strong evidence that cigarette smoking and dieting behaviors are positively associated [10].

Not surprisingly, the utilization of cigarettes and other tobacco products for weight control has potentially detrimental health effects on adolescents. Initiating smoking for weight control, and for those who currently smoke and fear cessation-related weight gain, continuing to smoke can lead to increased risk for cardiovascular and respiratory diseases and cancer [2]. Combined, smoking and diet/activity factors are the top leading causes of preventable death in the U.S. [11]. Smoking while overweight increases the risk for type 2 diabetes, hypertension, and poor lung functioning [12].

Evidence linking specific dieting behaviors to smoking is more variable with some studies showing unique ties between certain dieting behaviors (amphetamine/diet pill use; purging) and smoking, and others revealing the opposite (see [10] for a review). For example, adolescents who utilized purging dieting behaviors were more likely to report smoking compared to non-purging dieters and non-dieting adolescents, and dieters, in turn, reported greater cigarette smoking than non-dieters [13]. Gender differences when examining patterns of UDBs and associated covariates have been investigated more consistently. Among females, the association between UDBs and smoking appears to be the strongest [10, 14].

UDBs, like smoking, are associated with other risk-taking behaviors such as alcohol and drug use, delinquent behaviors, risky sexual behaviors [9, 14] and may induce other physical and psychological health consequences such as development of a clinical eating disorder [15]. Yet, little is known about the association between other types of tobacco use and UDBs. Of particular interest may be cigar smoking, a combusted tobacco product class with similar health effects as cigarette smoking [16, 17] and rising consumption patterns over the past decade [2, 18]. To our knowledge, only one study has examined cigar smoking in the context of weight control strategies [19]. Results suggested that cigar smoking and cigarette smoking were more prevalent among individuals not in weight control treatment. If like cigarettes, cigars and other alternative tobacco products are being used a weight control method or are positively associated with unhealthy dieting, awareness will be important for informing tobacco use prevention interventions as well as encouraging healthy eating behaviors in adolescence.

We know of no other published work that has examined other types of tobacco use in relationship to UDBs in an adolescent sample. The present study aimed to describe the prevalence of UDBs in a U.S.-based state sample of adolescents and examine whether the number of UDBs endorsed was associated with a variety of tobacco use behaviors among other psychosocial and behavioral characteristics.

## Methods

### Participants

This study utilized publically available de-identified secondary data from a representative sample of 6935 students aged approximately 12–18 years from the 2013 Virginia Youth Survey (VYS). Data for the current analysis were stratified by gender (missing for 32 individuals) and then by UDB endorsement status (missing at least one item among 123 females and 197 males); analyses included a total of 3501 female and 3402 male respondents (total *n* = 6903).

### Instrumentation

Participants answered questions related to demographics, tobacco product use, weight and dieting behaviors, psychosocial factors, and physical activity. As noted in each sub-section below, many item scales were collapsed or recoded. This strategy was utilized due to the low frequencies of individuals who engaged in UDBs which limited the use of response categories available.

#### Demographics

Age was assessed on a 7-point scale from 12 years old or younger to 18 years or older and was recoded to *less than 14* or *14 years and older* for the current analyses. Race/ethnicity categories included American Indian or Alaska Native, Asian, Black or African American, Native Hawaiian or Other Pacific Islander, Hispanic or Latino, Multiple races and/or ethnicities, and White. For the current analyses, the categories other than White (Non-Hispanic; NH) and Black or African American (NH) were subsumed under one category of “Other.”

#### Tobacco product use

Participants reported whether or not they ever used cigarettes (“even one or two puffs”; no/yes) and past 30-day cigarette use in days on a 7-point scale (0 days, 1 or 2 days, 3 to 5 days, 6 to 9 days, 10 to 19 days, 20 to 29 days, and all 30 days). For the current analyses, past 30-day cigarette use was recoded as no (0 days) or yes (1 or more days). Past 30-day alternative tobacco product use was assessed on the same 7-point scale as cigarette use with the following questions: “During the past 30 days, on how many days did you smoke cigars, cigarillos, or little cigars?” and “During the past 30 days, on how many days did you use chewing tobacco, snuff, or dip, such as Redman, Levi Garrett, Beechnut, Skoal, Skoal Bandits, or Copenhagen?” For the current analyses, cigar use and smokeless tobacco use were recoded as no (0 days) or yes (1 or more days).

#### Weight and unhealthy dieting behaviors

Participants were asked their height and weight in inches and pounds, respectively. Body mass index (BMI) was calculated and categorized based on age- and gender-specific percentiles [20]. BMI-for-age percentiles were then categorized as underweight (<5%), normal weight (≥5% to < 85%), overweight (≥85% to <95%), and obese (≥95%). Participants were asked what they were trying to do about their weight (weight intention; lose weight, gain weight, maintain, nothing). For the current analyses, the weight intention variable was recoded to maintain or nothing, lose weight, and gain weight. Participants also reported on whether they engaged in three UDBs in the past 30 days: 1) “During the past 30 days, did you go without eating for 24 h or more (also called fasting) to lose weight or to keep from gaining weight?” (no/yes; fasting), 2) “During the past 30 days, did you take any diet pills, powders, or liquids without a doctor's advice to lose weight or to keep from gaining weight? (Do not count meal replacement products such as Slim Fast.)” (no/yes; diet pills), and 3) “During the past 30 days, did you vomit or take laxatives to lose weight or to keep from gaining weight?” (no/yes, purging). A variable was created to assess the number of unhealthy dieting behaviors endorsed. This variable was coded on a 3-point scale from no UDBs (0), only one UDB (1), and 2 or more UDBS (combination of two or more UDBs; 2+).

#### Psychosocial factors

Participants reported whether they thought smokers had more friends on a 4-point scale: *definitely yes*, probably yes, probably not, and definitely not. This was recoded to definitely or probably not and definitely or probably yes. Additionally, adolescents were asked whether they were teased because of their weight in the past 12 months (no/yes).

#### Physical activity

Participants responded to a question about their physical activity during the past 7 days for which they were physically active for at least 60 min per day. Responses ranged on a 7-point scale from 0, 1, 2, 3, 4, 5, 6, to 7 days and were recoded based on other epidemiological studies [21]. Categories include no physical activity (0 days), low (1–2 days), moderate (3–5 days), and high (6–7 days).

### Procedure

The VYS is an ongoing statewide monitoring and surveillance survey of tobacco use and other health behaviors funded by the U.S. Centers for Disease Control and Prevention, the Department of Health in collaboration with the Virginia Foundation for Healthy Youth, with support from the Department of Education [4]. Description of the survey and methodology used are available elsewhere [22]. In brief, a two-stage cluster sample design of schools and classes is used to obtain a representative student sample in grades 9–12. The first stage of sampling involves choosing schools with probability proportional to enrollment, and during the second stage of sampling, intact classes are selected randomly. Weighting is used to adjust for non-response bias and the distribution of students by grade, sex, race/ethnicity in each state (i.e., geographic region sampled). The weight is applied to each student; weighted estimates are representative of all students in grades 9–12 in Virginia.

### Data analysis

Descriptive statistics were used to examine the sample in terms of endorsement of UDBs: none, one, and two or more behaviors. Per Youth Risk Behavior Surveillance System [23] guidelines, data were weighted by stratum, primary sampling unit, and individual weight and used the WR (with replacement at first stage) method. Weighted bivariate analyses for each predictor variable by UDBs were examined separately by gender. Predictor variables included age, race/ethnicity, ever smoking cigarettes, past 30-day cigarettes use, past 30-day cigar use, past 30-day smokeless tobacco use, weight intention, teased because of weight, belief that smokers have more friends, and physical activity. Weighted multinomial multivariate logistic regression analyses separately by gender were computed using Stata 13 Version (StataCorp, College Station, TX). Reporting no use of any UDB was used as the referent group in all logistic models (none vs. one UDB endorsed; none vs. two or more UDBs endorsed). Gender stratification was performed due to the strong evidence of gender-specific patterns in relationships between smoking behaviors and weight concern behaviors [10].

## Results

### Overall sample

Table 1 shows the frequency and weighted percentages of adolescents who were engaged in none, one, or two or more UDBs. Overall, 15.8% of the representative sample engaged in at least one UDB (*n* = 1163). Across the entire sample, the most prevalent UDB was fasting (14.2%) followed by purging (7.0%) and diet pill use (6.1%). Individuals who endorsed a single UDB (fasting-only) or a unique combination of UDBs (fasting and diet pill use) were less common. Across males and females, 6.9% reported fasting-only while only 1.6% and 1.5% reported diet pill use-only and purging behaviors-only, respectively. Unique combinations of UDBs ranged from 0.4% (diet pill use and purging) to 2.2% (all three behaviors). When examined by gender and UDB status, about 12.6% of females engaged in one type of UDB and 7.6% in two or more types of UDBs. For males, 7.3% engaged in one and 3.9% in two or more UDBs.

**Table 1:** Weighted percentage distribution of covariates by unhealthy dieting behaviors among female and male adolescents

	Females						Males					
Characteristics	n	%	0 UDBs	1 UDB	2+ UDBs	*p*	n	%	0 UDBs	1 UDB	2+ UDBs	*p*
Overall n	3501	100	2617	491	270		3402	100	2803	268	134	
Age												
< 14	813	20%	20%	22%	16%	0.305	712	19%	20%	18%	11%	0.097
≥ 14	2564	80%	80%	78%	84%		2490	81%	80%	82%	89%	
Race/ethnicity												
White NH	1750	56%	57%	48%	65%	**0.002**	1566	54%	55%	46%	53%	0.248
Black NH	673	23%	23%	27%	11%		657	23%	23%	27%	23%	
Other	891	21%	20%	26%	24%		913	23%	22%	27%	25%	
Ever smoke cigarettes												
No	2143	67%	73%	48%	35%	**<0.001**	1933	64%	67%	44%	26%	**<0.001**
Yes	1112	33%	27%	52%	65%		1104	36%	33%	56%	74%	
Past 30-day cigarette use												
No	2927	90%	93%	82%	73%	**<0.001**	2676	89%	90%	82%	70%	**<0.001**
Yes	331	10%	7%	18%	27%		359	11%	10%	18%	30%	
Past 30-day smokeless use												
No	3283	98%	98%	97%	93%	**<0.001**	2818	88%	90%	80%	68%	**<0.001**
Yes	90	2%	2%	3%	7%		377	12%	10%	20%	32%	
Past 30-day cigar use												
No	3118	93%	94%	88%	84%	**<0.001**	2794	87%	89%	79%	71%	**<0.001**
Yes	255	8%	6%	12%	16%		401	13%	11%	21%	29%	
Smokers have more friends												
No	2452	77%	80%	61%	65%	**<0.001**	2199	71%	74%	58%	37%	**<0.001**
Yes	799	24%	20%	39%	35%		880	29%	26%	42%	63%	
Weight intentions												
Nothing/maintain weight	1205	36%	41%	19%	10%	**<0.001**	1370	43%	46%	29%	17%	**<0.001**
Lose weight	1907	57%	51%	75%	87%		1005	30%	27%	54%	63%	
Gain weight	248	7%	8%	6%	4%		825	26%	27%	17%	20%	
BMI												
Underweight	52	2%	2%	1%	1%	**<0.001**	92	3%	3%	3%	1%	**0.001**
Normal weight	2241	72%	75%	61%	62%		1900	64%	65%	56%	53%	
Overweight	526	16%	15%	21%	19%		522	17%	17%	18%	15%	
Obese	373	11%	9%	17%	18%		511	16%	14%	23%	30%	
Teased about weight												
No	2178	67%	73%	52%	32%	**<0.001**	2415	77%	78%	69%	63%	**0.001**
Yes	1173	33%	27%	48%	68%		767	23%	22%	31%	38%	
Physical activity												
None	657	20%	19%	26%	20%	0.123	351	10%	9%	17%	40%	**<0.001**
Low	684	21%	21%	20%	21%		432	14%	13%	23%	25%	
Moderate	1172	37%	38%	31%	41%		1052	35%	37%	31%	13%	
High	754	22%	22%	23%	18%		1254	40%	42%	29%	21%	

*Note.* Bolded *p*-values were significantly different by UDB statu s (*p* < 0.05)

Participants could refuse questions or respond “I don’t know”

*UDB* unhealthy dieting behavior, *NH* non-hispanic, *BMI* body mass index

### Bivariate analyses

Bivariate analyses indicated significant differences by UDB status among females for race/ethnicity (*p* = 0.002) with proportions indicating more individuals identified as Black NH or Other in the one UDB group and White NH in the two or more UDB group relative to those who endorsed no UDB use. Individuals who endorsed UDB use were proportionally more frequent relative to those who did not for all tobacco use items: ever cigarette smoking, past 30-day cigarette use, past 30-day smokeless tobacco use, past 30-day cigar use (all *p*s < 0.001). Perception items indicated that UDB use groups were proportionally more likely to perceive smokers have more friends (smokers have more friends; *p* < 0.001), have greater weight loss intentions (weight intentions; *p* < 0.001), have BMI indicative of overweight/obese status (BMI; *p* < 0.001), and report being teased because of weight (teased about weight; *p* < 0.001). Similar findings were observed for males except for race/ethnicity by UDB status (*p* = 0.248) and the observation of a significant association for physical activity (*p* < 0.001) with proportionally fewer individuals with UDB use reporting higher levels of physical activity relative to those with no UDB use.

### Multinomial multivariate analyses

The multinomial multivariate model included all variables examined in the bivariate analyses. Tables 2 and 3 present the weighted multinomial logistic regression results for the psychosocial and behavioral risk factors of UDB status for female and male adolescents, respectively.

**Table 2:** Multinomial logistic regression results for female adolescents

	0 vs. 1 UDB			0 vs. 2+ UDBs		
	AOR	95% CI		AOR	95% CI	
Age						
< 14	Ref			Ref		
≥ 14 years	0.93	0.66	1.30	1.34	0.85	2.10
Race/ethnicity						
White NH	Ref			Ref		
Black NH	1.00	0.72	1.39	**0.35**	**0.17**	**0.73**
Other	1.36	0.96	1.93	0.98	0.59	1.63
Ever smoke cigarettes						
No	Ref			Ref		
Yes	**2.06**	**1.38**	**3.08**	**2.58**	**1.65**	**4.02**
Past 30-day cigarette use						
No	Ref			Ref		
Yes	1.56	0.97	2.51	**1.93**	**1.03**	**3.62**
Past 30-day smokeless use						
No	Ref			Ref		
Yes	0.41	0.16	1.06	0.72	0.31	1.66
Past 30-day cigar use						
No	Ref			Ref		
Yes	**1.64**	**1.08**	**2.50**	1.59	0.84	3.03
Smokers have more friends						
No	Ref			Ref		
Yes	**1.90**	**1.39**	**2.59**	1.46	0.92	2.30
Weight intentions						
Nothing/maintain weight	Ref			Ref		
Lose weight	**3.24**	**2.06**	**5.11**	**6.59**	**3.09**	**14.08**
Gain weight	1.01	0.43	2.38	1.30	0.48	3.48
BMI						
Under/normal weight^a^	Ref			Ref		
Overweight	1.09	0.75	1.59	0.98	0.61	1.60
Obese	1.36	0.90	2.05	1.32	0.78	2.24
Teased about weight						
No	Ref			Ref		
Yes	**2.24**	**1.71**	**2.93**	**5.08**	**3.25**	**7.92**
Physical activity						
None	Ref			Ref		
Low	**0.62**	**0.39**	**0.98**	0.80	0.37	1.71
Moderate	**0.65**	**0.44**	**0.97**	1.25	0.69	2.25
High	1.00	0.65	1.55	1.08	0.58	1.99

*Note.* Bolded cells were significantly associated (*p* < 0.05)

*UDBs* unhealthy dieting behaviors, *NH* non-hispanic, *BMI* body mass index, *AOR* adjusted odds ratio, *CI* confidence interval, *Ref* reference group

^a^Due to low cell counts, underweight and normal weight were combined as the reference group

**Table 3:** Multinomial logistic regression results for male adolescents

	0 vs. 1 UDB			0 vs. 2+ UDBs		
	AOR	95% CI		AOR	95% CI	
Age						
< 14 years	Ref			Ref		
14 years or older	0.86	0.47	1.57	**2.38**	**1.17**	**4.81**
Race/ethnicity						
White NH	Ref			Ref		
Black NH	1.47	0.94	2.30	0.61	0.25	1.50
Other	1.06	0.58	1.93	0.62	0.34	1.14
Ever smoke cigarettes						
No	Ref			Ref		
Yes	**2.11**	**1.29**	**3.46**	**4.14**	**1.86**	**9.22**
Past 30-day cigarette use						
No	Ref			Ref		
Yes	0.63	0.37	1.07	0.81	0.29	2.28
Past 30-day smokeless use						
No	Ref			Ref		
Yes	1.40	0.87	2.27	0.79	0.27	2.34
Past 30-day cigar use						
No	Ref			Ref		
Yes	**2.04**	**1.09**	**3.79**	1.59	0.62	4.08
Smokers have more friends						
No	Ref			Ref		
Yes	**1.57**	**1.09**	**2.25**	**2.54**	**1.42**	**4.54**
Weight intentions						
Nothing/maintain weight	Ref			Ref		
Lose weight	**3.38**	**2.30**	**4.97**	**5.66**	**1.64**	**19.46**
Gain weight	0.80	0.41	1.57	2.00	0.71	5.64
BMI						
Under/normal weight^a^	Ref			Ref		
Overweight	0.97	0.61	1.54	0.79	0.36	1.71
Obese	1.04	0.65	1.67	1.18	0.29	4.77
Teased about weight						
No	Ref			Ref		
Yes	1.37	0.85	2.23	1.22	0.53	2.80
Physical activity						
None	Ref			Ref		
Low	0.70	0.38	1.30	**0.34**	**0.15**	**0.78**
Moderate	**0.46**	**0.26**	**0.79**	**0.07**	**0.03**	**0.18**
High	**0.33**	**0.19**	**0.55**	**0.17**	**0.07**	**0.42**

Bolded cells were significantly associated (*p* < 0.05)

*Note. UDBs* unhealthy dieting behaviors, *NH* non-hispanic, *BMI* body mass index, *AOR* adjusted odds ratio, *CI* confidence interval, *Ref* reference group

^a^Due to low cell counts, underweight and normal weight were combined as the reference group

#### Females

For females across models, there were no significant associations for age, past 30-day smokeless tobacco use and BMI. Ever smoking cigarettes, past 30-day cigar use, perceiving smokers to have more friends, trying to lose weight versus nothing/maintaining, and being teased about weight all had significant positive associations with engaging in one UDB relative to none. There was not a significant association for race/ethnicity or past 30-day cigarette use for individuals who engaged in one UDB relative to none. Relative to those reported no physical activity, individuals who reported low to moderate physical activity had decreased odds of reporting one UDB. Identifying as Black NH race/ethnicity was negatively associated relative to White NH race/ethnicity among those who endorsed two or more UDBs relative to none. For tobacco use, ever cigarette use and past 30-day cigarette use were positively associated with reporting two or more UDBs relative to none. Additionally, trying to lose weight and being teased because of weight were positively associated with engaging in two or more UDBs relative to none.

#### Males

For males across models, there were no significant associations for race/ethnicity, past 30-day cigarette use, past 30-day smokeless tobacco use, BMI, and teased about weight. Ever cigarette smoking, past 30-day cigar use, perceiving smokers have more friends, and trying to lose weight versus maintain were all positively associated with engaging in one UDB relative to none. Moderate and high physical activity relative to none were negatively associated with engaging in one UDB relative to none. For two or more UDBs, older age, ever cigarette smoking, perceiving smokers have more friends, and trying to lose weight persisted as positive associations as well as negative associations with engaging in physical activity relative to none.

## Discussion

In the current representative Virginia adolescent sample, nearly 16% of adolescents engaged in at least one UDB. In both females and males, ever cigarette smoking, past 30-day cigar use, and the perception that smokers have more friends displayed the strongest tobacco-related patterns of association with UDB engagement in relative isolation (one UDB) as well as in combination (two or more UDBs). These overall rates of UDB engagement are comparable to past representative student samples [7, 24]. Previous studies have identified that smoking and UDBs are positively associated [14, 24]; however, to our knowledge, this is the first study to examine these specific associations in other tobacco products and find that past 30-day cigar use is also positively associated with the use of at least 1 UBD in both males and females.

The association between cigar use and UDB engagement is particularly important for adolescents in Virginia given that over 11% of the current sample reported past-30 day cigar use at rates comparable to current cigarette smoking [2]. Youth may be especially vulnerable to these alternative products due to the availability of flavored cigar products [25] and lower/differential tax rates at the federal and state level in the U.S. [26]. The association between engaging in a single UDB and cigar smoking indicates a possible sensitive period for intervention on cigar use and UDBs. Future studies may consider redefining UDB frequency to discern the qualitative differences between those who use one versus two or more UDBs to inform these results. Additionally, given that past month electronic cigarette use now exceeds rates of all other types of tobacco use [2], future studies may seek to address this alternative method of nicotine delivery in relation to risk for UDB engagement as well.

The current findings suggest that both male and female adolescents who have ever smoked cigarettes and perceive smokers to have more friends engage in more UDBs. This is consistent with findings that experimentation with cigarettes has been positively related to weight control behaviors [14]. However, contrary to previous studies that identified an association between current cigarette use and past use of unhealthy weight control behaviors [24] in the multinominal models past 30-day cigarette use in this study was positively associated only among females who endorsed two or more UDBs and not at all among males. Tomeo et al. [14] indicated that weight control behaviors, such as UDBs in the current study, might be associated with tobacco initiation rather than something that established smokers initiate. Cavallo et al. [7] suggested that adolescents who experiment with cigarettes may eventually rely on smoking for weight control as well as engage in UDBs. This evidence in combination with the current results highlights the ambiguity regarding the causal timeline for smoking initiation for weight control and weight intentions among adolescents.

One of the strongest associations, trying to lose weight and UDBs, was observed for both UDB endorsement categories and between both genders, similar to Johnson et al. [24]. Consistent with this finding, Tomeo et al. [14] found that adolescents contemplating and experimenting with smoking cigarettes were more likely to identify as overweight compared to those not contemplating cigarette smoking. Interestingly, BMI was not significantly associated with UDB engagement in either gender in one sample [14]; however, BMI has been associated with smoking for weight control among males but not female adolescent smokers [27]. It may be that perceiving a need for weight loss rather than objective measures (i.e., BMI) explains the association between trying to lose weight and UDBs.

Findings that were unique between genders suggested that among females, identifying as Black NH was a negatively associated with engaging in two or more UDBs. A lack of association among Black females for smoking for weight control and weight intentions has also been observed in another sample [28]. Taken together, these data may reflect the low incidence of UDBs among Black females as well as potentially unique set of predictors for UDB incidence among this group. However, among males, there were no associations between race/ethnic group and UDB endorsement. Engagement with two or more UDBs was positively associated with older age among males but not females suggesting that females may be similar risk for UDB engagement between the age groups analyzed. Additionally, the association between UDBs and being teased about weight was present for both categories of UDB engagement among females, but not males, which may indicate the importance of social perception on weight control behaviors for females.

Given that nearly 16% of the current sample of adolescents in 2013 engaged in at least one UDB in the past 30 days, which were linked to lifetime cigarette smoking, current cigar smoking, and other social factors surrounding weight and smoking, the findings add the current evidence and support the inclusion of all tobacco products in the prevention of problematic eating behaviors among adolescents, and risk taking prevention more generally. Implications for prevention in school and community settings that serve adolescents aged 14–18 such as in the current sample should include the impact of perceived social norms of tobacco use and weight control by utilizing peer-to-peer resources and anti-bullying policies and/or interventions with a focus on anti-weight-related bullying. Moreover, given the co-occurrence of these risk behaviors, anti-smoking campaigns should incorporate information on cigar/cigarillo/little cigar smoking. Primary prevention campaigns for adolescent eating disorders have been shown to actually *increase* eating disorder behavior [29]; therefore, it is not recommended to attempt to include weight concerns in current anti-smoking campaigns, as this inclusion may promote tobacco use. Thus, a secondary prevention approach may be more appropriate through screening current smokers for UDBs to identify those at risk and focusing on smoking cessation and weight concerns. Additionally, physical activity may serve a protective factor for unhealthy dieting practices, particularly among boys, highlighting the importance of physical education in schools, local communities, and public health campaigns which may prevent both tobacco use and UDBs. Health educators and healthcare providers should be aware that cigarette smoking and cigar smoking may be used for weight loss among youth and may affect their willingness to quit due to potential weight gain.

### Limitations and future directions

Due to the cross-sectional nature of the data, directionality of the findings cannot be determined. Longitudinal data would better clarify the causal association between social and behavioral risk factors for UDBs. Social desirability bias may have artificially underestimated tobacco use behaviors as well as UDBs among adolescents. These concerns may be outweighed by the anonymity of the survey, large sample size, and high response rate. Additionally, the reliability and validity of tobacco use self-reporting among adolescents has been found to be acceptable in previous examinations [30–32]. The results are limited to adolescents living in a specific geographic region of the U.S. (Virginia), specifically those aged approximately 12–18. Age may play an important role, particularly with regards to early initiation of smoking, which is predictive of adolescent and adulthood smoking [33, 34] and it may be important to examine younger ages in future studies. We were limited to the variables available in the database, which importantly only assessed past 30-day UDB engagement and tobacco use and did not include electronic cigarette or hookah use (waterpipe tobacco smoking) [35, 36]. In addition, inclusion and assessment of past year tobacco use may have strengthened the associations observed. We considered evaluating the predictors for each type of UDB behavior as in previous studies rather than collapsing across and using the number of UDBs endorsed alone as our outcome. Due to the low frequency of these individual behaviors, sample sizes were too small to ensure confidence in our findings. Future work that recruits specifically for individuals engaging in UDBs may elaborate on the specific isolated engagement as well as combinations of UDBs. Approximately 70% of the current sample had not ever smoked cigarettes, which limited our ability to make inferences about tobacco use. However, more people smoked cigars in this region compared to overall U.S. rates, which makes cigar smoking more relevant for youth in Virginia. Moreover, cigars are being used at similar if not greater rates than cigarettes among Virginia youth. Lastly, direct comparison of inferential statistics between female and male adolescents could address more specifically gender-specific differences for the predictors observed for UDB engagement, but due to the complex sampling design and multinomial models used comparative approaches were limited.

## Conclusions

The consistent relationships between lifetime cigarette smoking, social perceptions of smokers, and UDBs across genders highlight the interplay of these factors and their role in weight control behaviors as well as societal pressures for thinness across genders. These findings are the first to highlight an alternative tobacco product, cigars, as related to UDB engagement. Taken together this work should inform prevention efforts for tobacco use and UDBs as well as to give rise to healthy strategies for weight control such as physical activity and reinforcing a healthy body image. Pediatricians and school health programs should address the use of any tobacco/nicotine consumption methods for weight control at early stages in adolescence and young adulthood.
